# Impaired differentiation of small airway basal stem/progenitor cells in people living with HIV

**DOI:** 10.1038/s41598-022-06373-7

**Published:** 2022-02-22

**Authors:** Nancy P. Y. Chung, K. M. Faisal Khan, Mirko Andreoli, Robert J. Kaner, Sarah L. O’Beirne, Ronald G. Crystal

**Affiliations:** 1 Department of Genetic Medicine, Weill Cornell Medicine, 1300 York Avenue, Box 164, New York, NY 10065 USA; 2 Department of Medicine, Weill Cornell Medicine, New York, NY USA

**Keywords:** Cell biology, Diseases, Pathogenesis

## Abstract

With highly active anti-retroviral therapy (HAART), higher incidence of airway abnormalities is common in the HIV population consistent with the concept of accelerated lung “aging”. Our previous findings demonstrated that HIV induces human airway basal cells (BC) into destructive and inflammatory phenotypes. Since BC function as stem/progenitor cells of the small airway epithelium (SAE), responsible for self-renewal and differentiation of SAE, we hypothesized that BC from people living with HIV (PLWH) may have altered differentiation capacity that contribute to premature aging. The data demonstrates that BC from PLWH have impaired capacity to differentiate in vitro and senescent phenotypes including shortened telomeres, increased expression of β-galactosidase and cell cycle inhibitors, and mitochondrial dysfunction. In vitro studies demonstrated that BC senescence is partly due to adverse effects of HAART on BC. These findings provide an explanation for higher incidence of airway dysfunction and accelerated lung aging observed in PLWH.

## Introduction

With treatment of HIV infection with HAART, HIV individuals are living longer, but have an increased incidence of various aging-associated disorders, including accelerated development of chronic obstructive pulmonary disease (COPD)^1–8^. Clinical studies have demonstrated that significant airway abnormalities and decline in lung functions are found in PLWH^9–17^. Several studies have proposed that direct effect of HIV or its viral proteins, smoking, HAART therapy and chronic lung inflammation are contributing factors for the pathogenesis HIV-associated COPD^2,3,9,11,18–23^.

Human airway basal cells (BC) function as the stem/progenitor cells of the human small airway epithelium (SAE), responsible for maintaining the correct proportions of ciliated and secretory cells that function as the mucociliary component of lung host defense^24–26^. Our previous studies showed that HIV binding to BC induces the BC to acquire destructive and inflammatory phenotypes, with increased expression of matrix metalloproteinase-9 (MMP-9) and release of inflammatory mediators, contributing to the tissue destruction and chronic inflammation that characterizes the HIV^+^ lung^27,28^. In addition, BC from PLWH have increased release of inflammatory mediators^28^. Given these altered BC phenotypes, we hypothesized that BC from PLWH may also have altered differentiation capacity and senescence-associated phenotypes, resulting in the disordered airway epithelium observed in PLWH. To assess this hypothesis, we examined: (1) the differentiation capacity of airway epithelium derived from PLWH and HIV^–^ SAE BC; (2) biologic abnormalities of SAE BC from PLWH associated with altered differentiation and senescence; and (3) possible effects of HAART on the biology of normal small airway BC relevant to senescence and differentiation.

## Methods

### Small airway epithelium (SAE) BC from PLWH and HIV^−^ nonsmokers

All individuals enrolled in this study were assessed at the Department of Genetic Medicine Clinical Research Facility, using Weill Cornell Medicine Institutional Review Board-approved clinical protocols. Informed consent was obtained from each individual prior to this study. Individuals underwent an initial screening evaluation including history, complete physical exam, blood studies, urine analysis, chest X-ray, pulmonary function tests, and electrocardiogram. Individuals with any significant use of addictive drugs in the previous 6 months were excluded. Blood studies included a complete blood count, coagulation parameters, serum electrolytes, liver and kidney function tests, serum evaluation for human immunodeficiency virus antibodies, HIV-1 viral load, CD4 count and hepatitis profile (A, B, and C)^29^. Pulmonary function tests were carried out according to American Thoracic Society guidelines^30–33^. HIV^−^ nonsmokers (n = 4) and PLWH (n = 4) all had a normal screening evaluation, normal pulmonary function tests and chest X-ray, and a negative urine screen for smoking. Information of CD4 cell count, viral loads and HAART regimens were documented (see Table 1 for demographic details). All PLWH were nonsmokers receiving HAART and had no history of AIDS. Samples were collected before COVID pandemic.

**Table 1:** Demographics of Normal HIV^−^ Nonsmokers and PLWH.

Phenotype	ID#	Age/yr	Gender/ethnicity^1^	CD4 count cells/mm^3^	CD8 count cells/mm^3^	Viral load (copies/ml)	HAART therapy
PLWH	H-1	54	F (AA/Asian or Pacific Islander)	1106	1479	Not detected	Elvitegravir/Cobicistat/Emtricitabine/Tenofovir alafenamide (Genvoya)
	H-2	19	M (AA)	500	357	Not detected	Efavirenz,emtricitabine, and tenofovir disoproxil fumarate (Atripla)
	H-3	43	M (Hispanic)	1080	821	Not detected	Elvitegravir/Cobicistat/Emtricitabine/Tenofovir alafenamide (Genvoya/Valtrex)
	H-4	45	M (AA)	750	388	Not detected	Emtricitabine, rilpivirine, and tenofovir disoproxil fumarate (Complera)
HIV^−^	C-1	27	M (AA)	NA	NA	NA	NA
	C-2	47	M (AA)	NA	NA	NA	NA
	C-3	63	M (Hispanic)	NA	NA	NA	NA
	C-4	46	M (AA)	NA	NA	NA	NA

*F* female, *M* male, *AA* African-American, *NA* not applicable.

Small airway epithelial cells were collected by fiberoptic bronchoscopy by brushing as previously described^34–37^. After routine anesthesia, a 2 mm disposable brush (Wiltek Medical, Winston-Salem, NC) was inserted into the working channel of the bronchoscope and advanced to the airways distal to the orifice of the desired lobar bronchus. Small airway epithelial samples were obtained from the 10th to 12th order bronchi by sliding the brush back and forth on the epithelium 10–20 times at 8–10 sites. For each brush, after withdrawing from the bronchoscope, the cells were dislodged from the brush by flicking the brush tip in 5 ml of ice-cold PneumaCult-Ex Plus Medium (StemCell Technologies, Cambridge, MA). The airway epithelial cells collected by brushing were pelleted by centrifugation (250×*g*, 5 min) and disaggregated by resuspension in 0.05% trypsin-ethylenediaminetetraacetic acid (EDTA) for 5 min, 37 °C. Trypsinization was stopped by addition of 4-(2-hydroxyethyl)-1-piperazineethanesulfonic acid buffered saline (HEPES) buffered saline, (Lonza, Basel, Switzerland) supplemented with 15% fetal bovine serum (FBS; Life Technologies, CA), and the cells were again pelleted at 250 × g, 5 min. The pellet was resuspended with 5 ml of phosphate buffered saline, pH 7.4 (PBS), at 23 °C, then centrifuged at 250×*g*, 5 min. Following centrifugation, the cells (2.5 × 10^5^) were resuspended and plated in T25 flasks in 5 ml of PneumaCult-Ex Plus Medium and maintained in a humidified atmosphere of 5% CO_2_ at 37 °C. The next day, unattached cells were removed by changing the medium and thereafter, every 2 days. To passage the cells, the primary BC were seeded at a cell density of 3000 cells/cm^2^ in PneumaCult-Ex Plus Medium. The following day, the media was replaced with fresh medium and thereafter every 2 days.

### Air–liquid interface culture

BC differentiation was assessed using air–liquid interface (ALI) cultures. After the BC reached 70–80% confluence, the cells were trypsinized and seeded (density 1.5 × 10^5 ^cells/cm^2^) onto a 0.4 μm pore-sized Costar Transwells inserts (Corning) pre-coated with type IV collagen (Sigma) in PneumaCult™-Ex Plus Medium. After confluence, the basolateral medium was replaced with PneumaCult™-ALI Medium (StemCell Technologies), and the apical surface exposed to air (“ALI day 0”). The medium was changed every other day until ALI day 28, a time-point when normal BC generate a fully differentiated mucociliary airway epithelium. Epithelial barrier integrity was assessed by measuring Rt (Millicell-ERS epithelial ohmmeter, Millipore). At day 28, ALI culture inserts were washed with PBS for 2 times and fixed in 4% paraformaldehyde at room temperature for 30 min. After two PBS washes, fixed inserts in 70% ethanol Inc for low-melt paraffin embedding and sectioning then washed with PBS twice and embedded in low-melt paraffin block (Histoserv Inc, Germantown, MD). Five micrometer sections were cut for each ALI culture and stained with hematoxylin and eosin for histological analysis.

### Quantification of relative telomere length

Genomic DNA was isolated from BC using Gentra Puregene Kit (Qiagen). Five nanogram of genomic DNA serve as template and SYBR Green PCR Master Mix was used for reaction. SAE BC telomere length was determined by qPCR using Relative Human Telomere Length Quantification qPCR Assay Kit (ScienCell Research Laboratories)^38^. Amplification of telomeres and the single copy reference gene on human chromosome 17 (100 bp long) quantify telomere length as a relative T/S ratio (T = telomere, S = single copy gene) performed in duplicate for all samples.

### TaqMan gene expression

Differentiated airway epithelium derived from BC in ALI culture were homogenized in Trizol (Life Technologies). Total RNA was extracted using Trizol reagent (Invitrogen) and the aqueous phase was purified using an RNAEasy MinElute RNA purification kit (Qiagen). RNA concentration was determined using a NanoDrop ND-100 spectrophotometer (NanoDrop Technologies). First-strand cDNA was synthesized from 0.5 µg of total RNA using TaqMan Reverse Transcription Reagents with random hexamer as primer (Applied Biosystems). All samples were analyzed in triplicate at cDNA dilution of 1:10. The reactions were assessed using an Applied Biosystems Sequence Detection System 7500 and relative expression levels determined using the dCt method with 18S ribosomal RNA as an endogenous control^39^. Human BC are the adult stem/progenitor cells that differentiate into specialized airway epithelial ciliated and secretory cell during normal turnover and repair. To compare BC differentiation capacity of PLWH BC versus HIV^−^ BC, and the effect of HAART on BC differentiation, several differentiation-related genes including FOXJI and DNAI (ciliated gene markers), MUC5AC and MUC5B (secretory cell markers), SCGB1A1 (club cell marker) and TJP3 (tight junction marker) were assessed. To evaluate cellular senescence, expression of p16 and p21 (cell cycle inhibitors) were assessed. TaqMan probes were obtained from Applied Biosystems including FOXJ1 (Hs00230964_m1), DNAI1 (Hs00201755_m1), SCGB1A1 (Hs00171092_m1), MUC5AC (Hs01365616_m1); MUC5B (Hs00861588_m1), TJP3 (Hs00274276_m1), p16 (Hs00923894_m1) and p21 (Hs00355782_m1).

### β-Galactosidase expression

Senescence associated β-Gal (SA β-Gal) staining kit (Cell Signaling) was used to detect β-galactosidase (β-gal) activity. SA β-Gal catalyzes the hydrolysis of substrate, X-gal, which produces a blue color in senescent cells at pH6^40^. It has been widely used for quantification of senescent cells. Cells (5 × 10^4^) were plated on Type IV collagen-coated 24-well and cultured in complete PneumaCult-Ex Plus Medium for 2 days. After fixation, the cells were incubated with 300 μl 1 × β-gal staining solution at 37 °C overnight. β-Gal positive cells were quantified by light microscopy from 3 randomly chosen different fields of view.

### Assessment of mitochondria membrane potential

To assess mitochondrial dysfunction in BC, mitochondrial membrane potential kit (Cell Signaling) was used. Briefly, 10^4^ SAE-BCs were plated on 96-well and cultured in Ex-Plus media for 3 days. Cells were incubated with fluorescent cell permeable TMRE dye for 30 min, then washed with PBS three times. Fluorescence was measured at Ex 550 nm/Em at 580 nm.

### Immunofluorescence assessment of ALI sections

The airway epithelium in ALI culture was fixed in 4% paraformaldehyde/PBS for 20 min and washed with PBS for 3 times. The transwells were embedded in low melt temperature paraffin and cut into 5 µm sections (Histoserv Inc). The samples were first cleared in xylene and rehydrated with graded ethanol. To unmask the antigen, samples were steamed for 20 min in citrate buffer solution (Thermo Scientific), permeabilized with 0.1% TritionX-100 in PBS for 10 min and blocked with 10% normal goat serum for 45 min to reduce background staining. The samples were then stained overnight at 4 °C with antibodies to detect ciliated cells (β-tubulin IV, 5 μg/ml; MU178-UC; Biogenex) and secretory cells (MUC5AC, 5 μg/ml; sc-3367 and MUC5B, 4 μg/ml; sc-20119; Santa Cruz). Mouse IgG (control for MUC5AC and β-tubulin IV) and rabbit IgG (control for MUC5B) were used as negative controls (Jackson ImmunoResearch Lab). Alexa Fluor 555 Goat Anti-mouse IgG (A-21422; Invitrogen) and Alexa Fluor 555 Goat Anti-rabbit IgG (A-11035; Invitrogen) labeled secondary antibodies were used to visualize antibody binding. The cells were stained with DAPI to identify cell nuclei and subsequently mounted using ProLong Gold antifade reagent (Invitrogen). Fluorescence microscopy was performed using a Zeiss Axioplan body microscope with either a 40× or 100× lens and evaluated using a Zeiss high resolution monochrome camera.

### Assessment of HAART induction of SAE BC cytotoxicity

A lactate dehydrogenase (LDH) colorimetric assay (Thermo Scientific, Rockford, IL) was used to assess the effect of the HAART drugs emtricitabine (FTC) and tenofovir disoproxil fumarate (TDF; Selleckchem, Houston, TX) on normal BC. BC (10^4^/100 µl) were plated in 96 wells and incubated overnight. On the next day, cells were incubated with FTC, TDF or in combination at 0.5–10 μM for 48 h. Each treatment group was assessed in triplicate. Spontaneous LDH activity controls compared to maximum LDH activity control was used to assess drug-induced toxicity. After 72 h, 10 µl of water and lysis buffer were added to the spontaneous LDH activity controls (water) and maximum LDH activity controls (10 × Lysis Buffer) respectively and incubated at 37 °C for 45 min. Following that, 50 µl of each sample media (i.e. media from untreated BC, DMSO-treated, HAART-treated BC, spontaneous LDH activity controls and maximum LDH activity controls) were transferred to a 96-well flat-bottom plate and mixed with the reaction mixture. After 30 min incubation at 23 °C, reactions were stopped by adding stop solution. Absorbance at 490 nm and 680 nm was measured using spectrophotometer to determine LDH activity. The percentage of cytotoxicity was calculated using the formula: [HAART-treated LDH activity − Spontaneous LDH activity]/[Maximum LDH activity − Spontaneous LDH activity] × 100.

### Effect of HAART on SAE BC and ALI

SAE BC from HIV^−^ nonsmokers were treated with FTC, TDF alone or in combination at 1 and 5 µM. DMSO (0.05%) was used as control. BC were plated in T25 flask at density of 3000 cells/cm^2^ and cultured in complete PneumaCult Ex-Plus medium containing HAART for 2 passages (passage 1: day 0–4; passage 2: day 5–14). Genomic DNA and total RNA were collected from each passage for assessment of telomere length and gene expression. For each passage, β-galactosidase expression and mitochondrial membrane potential of BC treated with HAART were measured as described above.

To study the effect of HAART on BC differentiation, ALI culture maintained in complete PneumaCult ALI medium containing HAART until day 28. ALI medium (with/without drugs) were replaced every 2 days. At day 28, genomic DNA and total RNA were collected for assessment of the BC. For analyses of BC differentiation capacity in DMSO-treated *vs* HAART-treated ALI, TaqMan PCR was performed for expression of genes specific for ciliated cells (FOXJ1), club cells (SCGB1A1), secretory cells (MUC5AC and MUC5B) and cell cycle inhibitors (p16 and p21).

### Statistical analysis

For ALI thickness analysis and number of nuclei, six measurements at different regions of each individual’s ALI section were made and the average thickness was quantified. Comparison of normal *vs* PLWH ALI sections (n = 4 for each phenotype) were calculated using unpaired two-tailed Student’s t tests with unequal variance. For TaqMan gene expression and telomere length analyses, triplicate was made from each individual and the mean of each individual was calculated. Comparison of normal *vs* PLWH ALI sections (n = 4 for each phenotype) were calculated using unpaired two-tailed Student’s t tests with unequal variance. For immunofluorescent analysis, fluorescent intensity was quantified using Image J software. Three measurements at different region of each individual’s ALI section were made and average fluorescent intensity/area of section in square pixel was calculated. Comparison of normal vs PLWH ALI sections (n = 4 for each phenotype) were calculated using unpaired two-tailed Student’s t tests with unequal variance. For mitochondrial membrane potential analyses in HAART-treated BC vs DMSO group and PLWH *vs* normal individual, quadruplicate was made for each HAART treatment or each individual. Average mitochondrial membrane potential was calculated for each HAART treatment groups or each individual. Comparison of normal *vs* PLWH ALI sections (n = 4 for each phenotype) or HAART-treated BC vs DMSO group were calculated using unpaired two-tailed Student’s t tests with unequal variance. For all the analyses, values of p < 0.05 were considered significant.

## Results

### Impaired proliferation/differentiation of BC from PLWH compared to HIV^−^ SAE BC

To eliminate the effect of smoking of any type on BC biology, all the studies were carried out in nonsmokers. SAE BC from age-matched healthy HIV^−^ nonsmokers (n = 4) and PLWH (n = 4) were assessed (Table 1). For PLWH, all were receiving HAART treatment with undetectable viral load.

**Figure 1:**
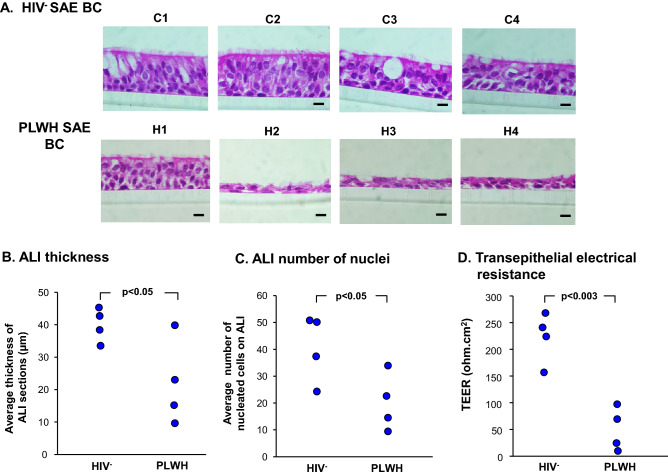
Evidence of impaired differentiation of PLWH SAE BC compared to HIV^−^ SAE BC. Purified primary BC from PLWH and HIV^−^ nonsmokers were cultured on ALI for 28 days. At day 28, the thickness of the cell layer was quantified in fixed sections at 6 points randomly chosen along the entire ALI section using ImageJ software, and the average thickness was quantified. (**A**) Differentiated epithelium from HIV^−^ SAE BC (C-1 to C-4; upper panel) and PLWH SAE BC (H-1 to H-4; lower panel). (**B**) Quantification of ALI thickness derived from BC of HIV^−^ nonsmokers and PLWH (n = 4 for each phenotype). (**C**) Quantification of nuclei number from the same samples as in panel (**B**). (**D**) Transepithelial electrical resistance of HIV^−^ and PLWH SAE BC derived ALI. Purified primary BCs were cultured on ALI for 28 days using standard conditions. Transepithelial electrical resistance (ohm cm^2^) of ALI derived from HIV^+^ nonsmokers and PLWH (n = 4 for each phenotype) were assessed at Day 28.

Primary basal cells purified from human SAE sampled by bronchoscopic brushing of the HIV^−^ and PLWH were assessed for the capacity to differentiate on ALI culture. At day 28, the thickness of the differentiated epithelium layer was quantified in hematoxylin and eosin-stained sections at 6 points randomly chosen along the entire ALI section using ImageJ software, and the average thickness of each airway epithelium quantified. Compared to normal HIV^−^ controls (C-1 to C-4), BC from PLWH (H-1 to H-4) generated a thinner epithelium with the exception of H-1 whose CD4 and CD8 cell counts were the highest (p < 0.05; Fig. 1A,B). The total number of nucleated cells in airway epithelium derived from PLWH BC was significantly reduced when compared to HIV^−^ epithelium (p < 0.05; Fig. 1C). Transepithelial electrical resistance, a measure of tight junctional barrier integrity, was determined at day 28 of ALI culture. ALI cultures derived from SAE BC of PLWH had significantly lower transepithelial electrical resistance compared with ALI derived from HIV^−^ nonsmokers (p < 0.003; Fig. 1D).

**Figure 2:**
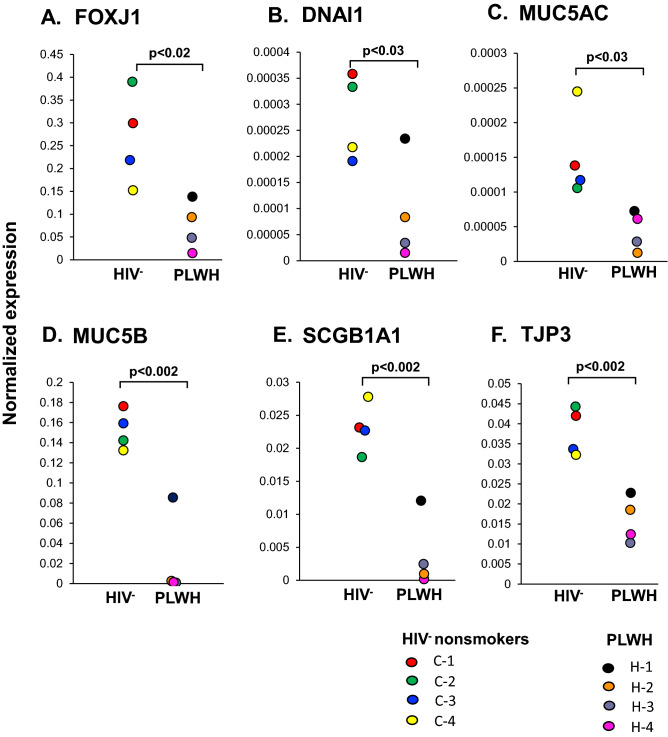
Disordered differentiation of ALI derived from PLWH SAE BC. Expression of differentiated-related genes in HIV^−^ and PLWH SAE BC-derived ALI. (**A**) FOXJ1(ciliated cells); (**B**) DNA1 (ciliated cells); (**C**) MUC5B (secretory cells); (**D**) SCGB1A1 (club cells) and (**E**) TJP3 (tight junction) assessed by Taqman PCR. The data were normalized to 18s RNA. Data shown are the mean of each individual from one representative of three independent experiments performed in triplicate. Each individual of HIV^−^ nonsmokers (C-1 to C-4) and PLWH (H-1 to H-4) was color-coded as indicated.

**Figure 3:**
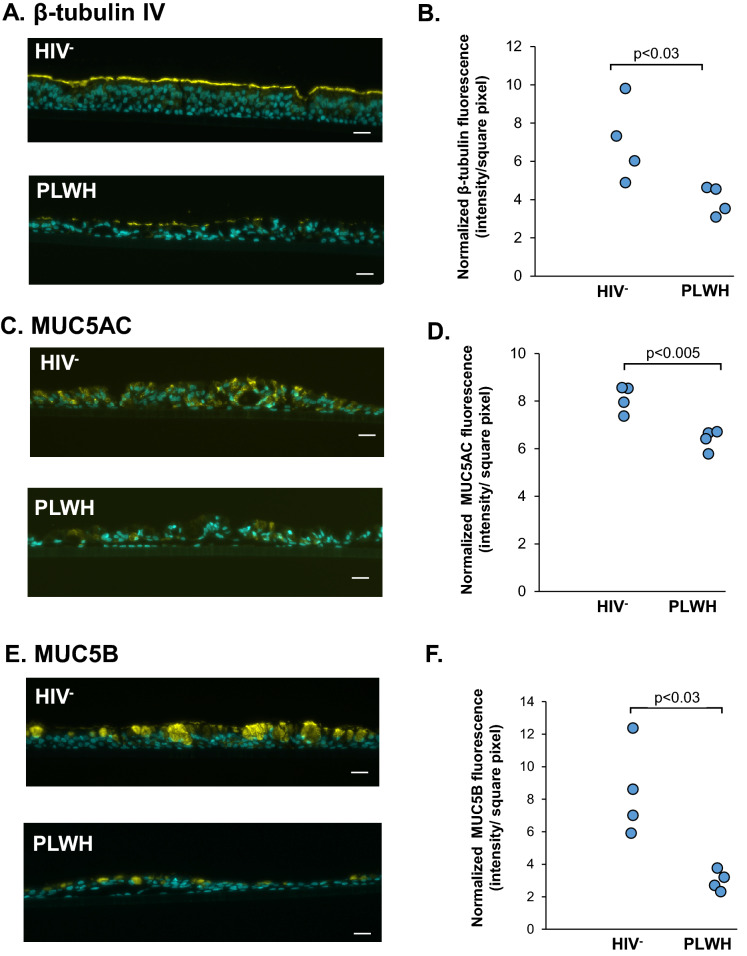
Decreased ciliogenesis, MUC5ACand MUC5B expression on ALI derived from PLWH. (**A**) Immunofluorescence of β-tubulin IV (ciliated cell markers, yellow) and nuclei (cyan) in HIV^−^ (upper panel) and PLWH SAE BC-derived airway epithelium (lower panel). Representative examples are shown. (**B**) Quantification of β-tubulin IV expression on ALI derived from HIV^−^ and PLWH SAE BC (n = 4 for each phenotype). (**C**) Immunofluorescence of MUC5AC (secretory cell marker, yellow) and nuclei (cyan) in HIV^−^ (upper panel) and PLWH SAE BC-derived airway epithelium (lower panel). Representative examples are shown. (**D**) Quantification of MUC5AC expression on ALI derived from HIV^−^ and PLWH SAE BC. (**E**) Immunofluorescence of MUC5b (secretory cell marker, yellow) and nuclei (cyan) in HIV^−^ (upper panel) and PLWH SAE BC-derived airway epithelium (lower panel). Representative examples are shown. (**F**) Quantification of MUC5B expression on ALI derived from HIV^−^ and PLWH SAE BC. Immunofluorescent intensity was quantified using ImageJ software and normalized with the area of ALI section as square pixel. Bar 20 µm.

The differentiation status of the ALI cultures from PLWH *vs* HIV^−^ nonsmokers that survived to day 28 was assessed by TaqMan gene expression analysis. Compared to HIV^−^ nonsmokers (C1–C4), expression of ciliated cell (FOXJ1 and DNAI, p < 0.02 and p < 0.03; Fig. 2A,B), secretory cell (MUC5AC and MUC5B, p < 0.03 and p < 0.002; Fig. 2C,D), club cell (SCGB1A1, p < 0.002; Fig. 2E) and tight junction (TJP3, p < 0.002; Fig. 2F) markers were downregulated in PLWH. (H1–H4). Immunofluorescent analysis of ciliated cell (β-tubulin IV) and secretory cell (MUC5AC and MUC5B) markers were performed to validate the gene expression data. Expression of β-tubulin IV was significantly lower in the ALI derived from PLWH compared to HIV^−^ nonsmokers (p < 0.03, Fig. 3A,B). Similarly, expression of MUC5AC (p < 0.005; Fig. 3C,D) and MUC5B (p < 0.03, and Fig. 3E,F) in ALI derived from PLWH was significantly lower than HIV^−^ ALI. Mouse IgG_1_ and rabbit IgG were as used as isotype controls for β-tubulin IV/MUC5AC and MUC5B, respectively. All ALI sections were stained negative (Supplemental Figs. 3, 4). Together, these data demonstrate that the ALI cultures from PLWH have an impaired differentiation phenotype with decreased number of ciliated and secretory cells.

### SAE-BC isolated from PLWH have senescent phenotypes

Since BC function as stem/progenitor cells to maintain correct populations of ciliated and secretory cells in airway epithelium, we investigated if any biologic abnormalities and altered phenotypes in BC from PLWH were associated with impaired BC differentiation capacity. To assess cellular senescent phenotypes in BC, several parameters including relative telomere length, expression of senescence-associated β-galactosidase, mitochondrial membrane potential and expression of cell-cycle inhibitors (p16 and p21) were examined.

**Figure 4:**
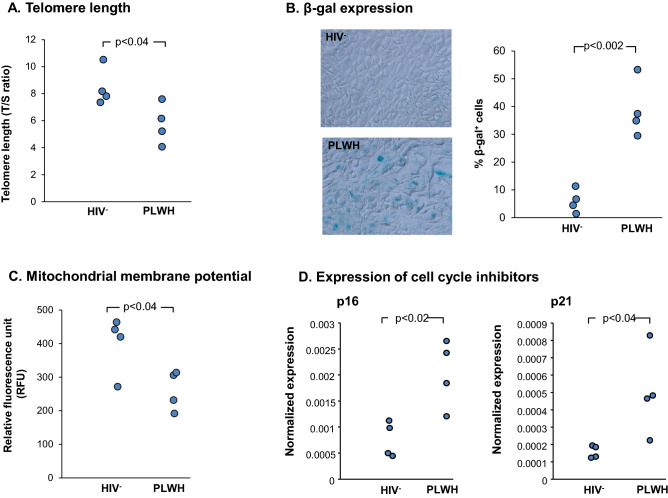
Biologic abnormalities of SAE BC from PLWH. (**A**) Telomere length of SAE BC of PLWH compared to HIV^−^ nonsmokers. Telomere length was assessed by qPCR of telomere repeat sequences (T/S ratio of telomere repeat copy number to single gene copy number). Data shown are the mean of each individual performed in triplicate. (**B**) Expression of β-galactosidase (β-gal) in BC isolated from HIV^−^ nonsmokers (upper panel) and PLWH (lower panel). Quantitative percentage of β-gal^+^ cells in HIV^−^ nonsmokers *vs* PLWH cultured in vitro. (**C**) Assessment of mitochondrial membrane potential in BC isolated from HIV^−^ nonsmokers and PLWH. BC were cultured for 3 days and incubated with fluorescent TMRE dye for 30 min to allow mitochondrial uptake. Fluorescent intensity was measured at with excitation at 550 nm and emission at 580 nm. Each data point represents the mean of each individual performed in triplicate. One representative of three independent experiments is shown. (**D**) Increased expression of cell cycle inhibitors p16 and p21 in ALI derived from SAE BC of HIV^−^ and HIV^+^ nonsmokers. BC from HIV^−^ nonsmokers and PLWH were cultured on ALI. TaqMan quantitative PCR analysis was performed for p16 and p21. The data were normalized to 18s RNA. Data shown are the mean of each individual from one representative of three independent experiments performed in triplicate.

Relevant to the pathogenesis of COPD, shortened telomeres trigger persistent activation of DNA damage response pathways resulting in cellular senescence^41^. Assessment of SAE BC from PLWH demonstrated shortened telomere length as compared to BC of HIV^−^ nonsmokers (T/S ratio in PLWH *vs* HIV^−^ nonsmoker: p < 0.04; Fig. 4A). In addition, BC from HIV^−^ and PLWH were grown on collagen-coated wells for 48 h and stained for β-galactosidase expression. BC isolated from PLWH demonstrated high levels of β-gal expression compared to BC from HIV^−^ nonsmokers (Fig. 4B). The percentage of β-gal^+^ cells from PLWH was significantly higher when compared to HIV^−^ nonsmoker (p < 0.002; Fig. 4B). In addition, BC from PLWH had decreased mitochondrial membrane potential compared to BC from HIV^−^ nonsmokers (p < 0.04; Fig. 4C). Assessment of BC from PLWH demonstrated increased expression of both p16 (p < 0.02; Fig. 4D) and p21 (p < 0.04; Fig. 4D) compared to HIV^−^ nonsmokers. Together, these findings support the concept that BC from PLWH are undergoing cellular senescence, resulting in reduced differentiation potential and disordered airway epithelium.

### HAART causes abnormalities in BC function

**Figure 5:**
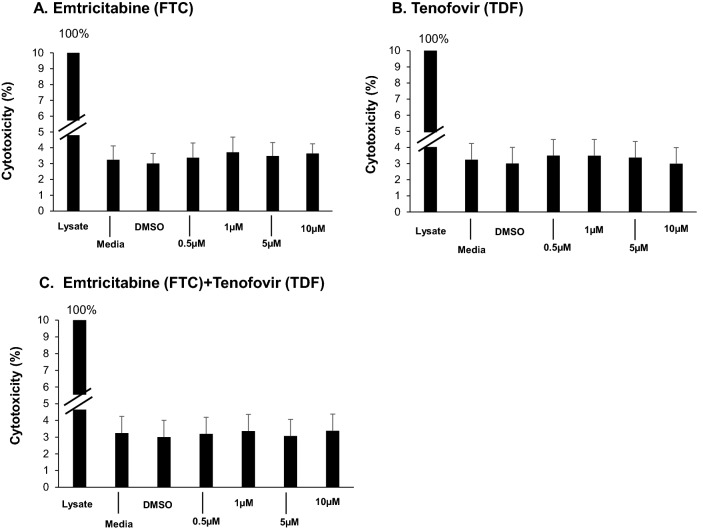
HAART drugs **(**FTC and TDF) do not evoke BC cytotoxicity. Normal HIV^−^ SAE BC were exposed to the drugs at the indicated concentrations either alone or in combination for 48 h. 0.1% DMSO was used as a solvent control. Supernatants were assessed for lactic dehydrogenase (LDH) activity. Triton X (1%) lysed cells serve as positive control (100%). Data shown are the mean from one representative of three independent experiments performed in triplicate.

In the context that BC of PLWH have evidence of premature aging compared to matched HIV^−^ individuals, we hypothesized that there may be adverse consequences of HAART on BC function and differentiation. Two commonly used HAART drugs, FTC and TDF were tested on BC. To ensure that there was no direct drug cytotoxicity on the BC, normal BC were exposed to the drugs either alone or in combination at in vivo relevant concentrations (0.5–10 µM) for 48 h. Supernatants were assessed for LDH cytotoxicity. At the concentrations used, FTC, TDF or both together did not induce toxicity compared to 0.1% DMSO (all < 5% of LDH cytotoxicity when compared to whole cell lysate control; Fig. 5).

**Figure 6:**
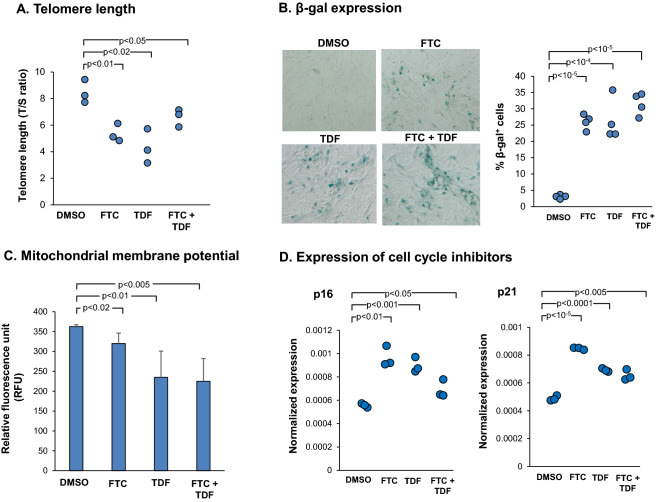
HAART drugs (FTC and TDF) induce biologic abnormalities in normal HIV^−^ SAE BC. (**A**) HIV^−^ SAE BC exposed to FTC and TDF have shorter telomeres. BC treated with HAART either alone or in combinations at 5 µM for 14 days (passage 2). Data shown represent telomere length (as T/S ratio) from cells at passage 2 from three separate experiments. (**B**) Exposure of normal HIV^−^ SAE BC to HAART increase expression of β-galactosidase. BC were incubated with FTC, TDF alone or in combination at 5 μM (passage 2). DMSO solvent (0.05%) served as a control. Increased number of β-gal^+^ cells was observed in BC treated with FTC, TDF and FTC + TDF as compared to DMSO. Percentage of β-gal^+^ cells in each treatment group was quantified. Data from 4 experiments are shown. (**C**) HAART decreased mitochondrial membrane potential in SAE BC. Cells were treated with HAART (passage 2) and incubated with fluorescent TMRE dye for 30 min to allow mitochondrial uptake. Fluorescent intensity was measured at with excitation at 550 nm and emission at 580 nm. Results shown represent the data from four individual experiments. (**D**) HAART upregulated expression of cell cycle inhibitors p16 and p21 in BC. Cells were treated with HAART either alone or in combinations at 5 µM (passage 2). Data shown represent expression of p16 and p21 from three separate experiments.

To further investigate possible effects of HAART drugs on BC, cells were treated with increasing dose of HAART (0.5–10 µM) and passaged two times (passage 1: day 0–4; passage 2: day 5–14). At passage 2, cells were assessed for senescence-associated markers including expression of β-galactosidase, mitochondrial membrane potential and cell cycle inhibitor p16. β-Galactosidase positive cells and p16 mRNA expression were increased in HAART-treated BC in a dose-dependent manner (Supplemental Fig. 1A,C). BC treated with increasing dose of HAART showed a dose-dependent reduction of mitochondrial membrane potential (Supplemental Fig. 1B). For further analysis, normal HIV^−^ BC were treated with FTC and TDF either alone or in combination at 5 µM. HAART-treated BC had shorter telomere length as compared to the DMSO control (all p < 0.05; Fig. 6A). BC were grown on collagen-coated plate in the presence of drugs and stained for β-galactosidase expression at passage 2. FTC and TDF-treated BC demonstrated higher levels of β-gal expression compared to the DMSO control (Fig. 6B). Increased percentage of β-gal^+^ cells was observed from cells treated with FTC (27%, p < 10^–5^), TDF (26.3%, p < 10^–4^) and FTC + TDF (31.5%, p < 10^–5^) when compared to the DMSO control (3%; Fig. 6B). Assessment of mitochondrial membrane potential demonstrated that HAART-treated BC at 5 µM had decreased mitochondrial membrane potential compared to DMSO (all p < 0.02 compared to DMSO control; Fig. 6C). Further, both FTC and TDF significantly up-regulated expression of cell cycle inhibitors p16 and p21 (all p < 0.05 compared to DMSO control; Fig. 6D).

To test if HAART removal could reverse the senescence phenotype, cells were passaged for additional 14 days in complete ExPlus medium without drugs after 14-day HAART treatment. Cells were harvested for p16 expression and β-galactosidase staining. After removal of HAART pressure, the number of β-gal^+^ cells in pre-HAART treated groups was significantly higher than DMSO control (all p < 0.05 compared to DMSO control; Supplemental Fig. 2A). Similarly, TaqMan PCR showed that p16 mRNA expression was increased in pre-HAART treated groups as compared to DMSO (all p < 0.05, Supplemental Fig. 2B). These findings suggest that HAART (either alone or in combination, at 1 and 5 µM) could not reverse the senescence phenotype.

**Figure 7:**
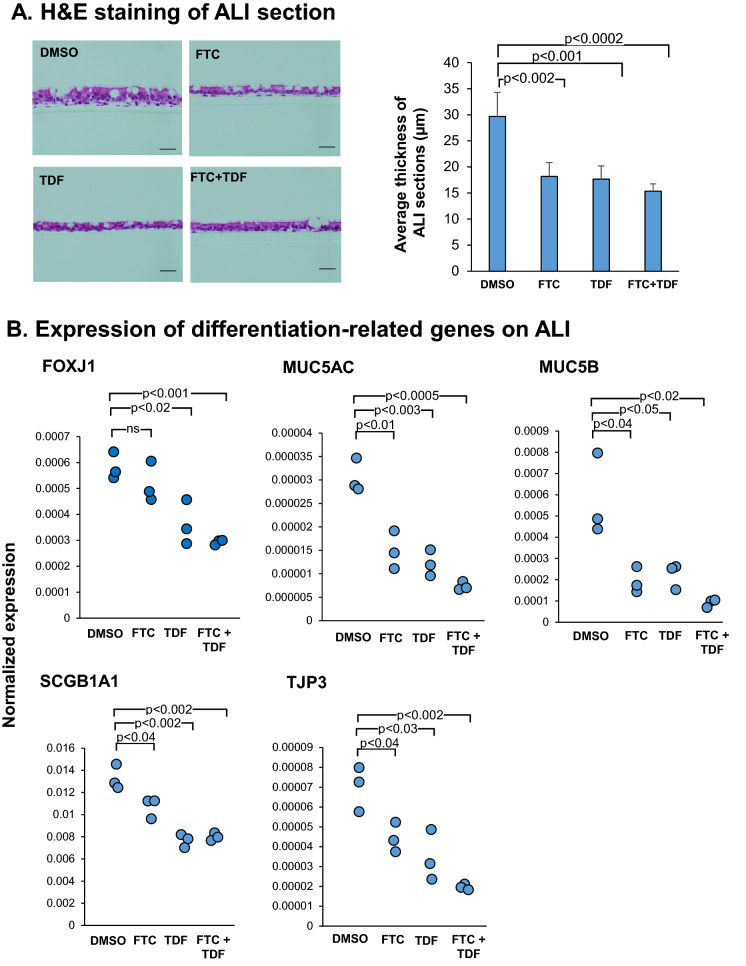
Effect of HAART on differentiation capacity of HIV^−^ SAE BC. (**A**) HAART treatment results in thinner epithelium. SAE BC were grown on ALI in the presence of FTC, TDF and FTC + TDF (all at 5 µM) for 28 days. DMSO solvent (0.05%) served as a control. Culture media containing HAART were replaced every 2 days. At day 28, ALI transwells were fixed in 4% paraformaldehyde and stained with hematoxylin and eosin. Bar 50 µm. (**B**) HAART downregulate expression of genes related to BC differentiation. Total RNA was extracted for assessment of differentiation-related markers including FOXJ1, TJP3, MUC5AC, MUC5B and SCGB1A1 by TaqMan quantitative PCR. Data from three separate experiments are shown.

We further examined the effect of HAART on BC differentiation. BC were grown on ALI in the presence of FTC, TDF or FTC + TDF for 28 days. DMSO served as a solvent control. Culture media containing FTC and TDF (5 µM) were replaced every 2 days. At day 28, the thickness of the differentiated epithelium layer from DMSO and drug-treated groups was quantified in hematoxylin and eosin-stained sections using ImageJ software. Exposure to the drugs resulted in suppression of differentiation with markedly thinner epithelium in FTC (18.2 ± 2.6 µm, p < 0.002), TDF (17.7 ± 2.5 µm, p < 0.001) and FTC + TDF (15.4 ± 1.4 µm, p < 0.0002; Fig. 7A) compared to DMSO control (29.7 ± 4.6 µm). We also assessed the status of differentiation-related genes of HAART-treated ALI cultures at Day 28. Compared to the DMSO control, HAART-treated ALI showed downregulation of FOXJ1 (p < 0.02 for TDF and FTC + TDF but not significant in FTC alone), MUC5AC (all p < 0.01), MUC5B (all p < 0.05), SCGB1A1 (all p < 0.04) and TJP3 (all p < 0.04) as assessed by TaqMan gene expression analysis (Fig. 7B). Taken together, these observations support the concept that HAART induces BC senescence and impairs BC differentiation.

## Discussion

With HAART therapy, the incidence of opportunistic infections and mortality has been markedly reduced^42^, but the treated HIV^+^ individuals have an increase of chronic aging-related disorders including COPD^2,5,7,43–47^. Several studies have shown the pulmonary/airway abnormalities observed in PLWH, including nonsmokers receiving HAART treatment^9–11,13–17^. PLWH on HAART therapy have chronic lung inflammation^48–55^, contributing to HIV-associated aging, lung injury and tissue damage^2,21–23,50,53,55,56^. Our recent studies demonstrate that HIV binding to airway BC increased secretion of MMP-9 and inflammatory mediators^27,28^ and BC from PLWH showed increased release of IL-8 IL-1β, ICAM-1 and GM-CSF^27,28^. These mediators and proteases such as MMPs are collectively known as senescence associated secretory phenotypes (SASP)^57,58^.

Based on these observations, we hypothesized that PLWH may have altered differentiation capacity and senescence-associated phenotypes, resulting in disordered airway epithelium. Our findings demonstrate that BC isolated from PLWH exhibit decreased capacity to differentiate normally and have increased p16 and p21 expression and signs of cellular senescence with shortened telomeres, increased expression of β-galactosidase and reduced mitochondrial membrane potential. Consistent with the observations of SAE BC obtained from PLWH, normal BC exposed to HAART (FTC and TDF) in vitro expressed senescence-related biomarkers and failed to form a normal differentiated epithelium.

### BC senescence contribute to accelerated lung aging in PLWH

In PLWH, accelerated lung aging is characterized by loss of lung function, chronic inflammation and airway abnormalities^9–15^. Cellular senescence is a hallmark of aging in the HIV^+^ population^59–61^ and accumulation of senescent cells is closely associated with age-related diseases^62,63^. Senescent cells are characterized by telomere shortening, increased expression of cell cycle inhibitors, mitochondrial dysfunction, secretion of proinflammatory cytokines and proteases (knows as SASP) and increased β-galactosidase level^58,60,64–68^.

Our study demonstrated that BC from PLWH exhibited senescent phenotypes, resulting in impaired differentiation and disordered airway epithelium. Consistent with our data, several studies have showed that significant decrease in telomere length is found in small airway epithelium and peripheral blood leukocytes of PLWH, suggesting accelerated aging in PLWH^69–72^. Cellular senescence is a contributing factor of diminished differentiation and stem cell exhaustion and linked to age-related disorders^64,73–78^. Senescent cells are terminally growth arrested, with up-regulation of cell cycle regulators such as p16 and p21 and poorly differentiated^73–75,77–79^. Using ALI culture, we found that decreased ciliogenesis was observed in poorly PLWH BC-derived differentiated airway epithelium. This is consistent with clinical data that HIV infection is associated with decreased mucociliary clearance, resulting in decreased host defense to pathogens^80,81^. Recent studies showed that lung epithelial integrity is disrupted by HIV^82^ and tracheobronchial mucociliary clearance is inhibited in the presence of HIV^83^. Our findings provide evidence that BC senescence results in abnormal airway differentiation and disordered epithelium in PLWH, causing diminished mucociliary and defense functions that in turns contributes to the risk of airway infection.

### BC abnormalities mediated by HAART

Prior studies have demonstrated that HAART such as emtricitabine (FTC) and tenofovir disoproxil fumarate (TDF) caused premature senescence associated with mitochondrial dysfunction and increased oxidative stress^59–61^. Analysis of bronchoalveolar fluid have shown that HAART drugs penetrate to the lung at 60 to 73% of plasma levels^84,85^. Extensive studies have shown that HAART drugs adversely affect mitochondrial functions, including inhibition of mitochondrial-specific DNA polymerase γ, causing mitochondrial damage by increasing oxidative stress and diminishing mitochondrial function^59,86–92^. In the lung, HAART causes mitochondrial damage in pulmonary arterial endothelial cells and lung fibroblasts^93,94^. Additionally, HAART such as TDF, FTC and azidothymidine suppress telomere activity and shortened telomere length in peripheral blood leukocytes^95–98^. Similar to BC, HAART has adverse effects on other lung cells. Recent study revealed that alveolar macrophages from HIV^−^ individuals receiving PrEP demonstrated an impaired epigenetic and transcriptional responsiveness to Mycobacterim tuberculosis^99^. Of interest, HAART also causes suppressive impact on microbiota diversity in the gut, which potentially causes intestinal dysbiosis and incompletely restore gut mucosal barrier dysfunction in patients^100–103^. PLWH have increased expression of cell cycle inhibitors, p16 and p21 in human vascular endothelial cells and adipocytes^104,105^. These studies support our observations that HAART induces senescent phenotypes in normal BC with decreased telomere length, increased cell cycle inhibitors and reduced mitochondrial membrane potential, suggesting that HAART could be one of the driving factors of accelerated aging in HIV^+^ lung.

As part of this study to assess small airway epithelium (SAE) basal stem/progenitor cell (BC) function in individuals infected with HIV, we observed that, despite clinically effective HAART therapy (undetectable HIV virus in blood, normal T cell levels), there was biologic dysfunction of BC, with evidence of aging, and abnormal differentiation and significantly decreased lung function compared to matched HIV^−^ individuals. Since PLWH had no evidence of active HIV infection, we assessed the hypothesis that anti-viral drugs might have an adverse effect on BC biology. Strikingly, the drugs (FTC and TDF) used in preexposure prophylaxis (PrEP), had adverse effects on BC function including shortened telomeres, increased expression of β-galactosidase, cell cycle inhibitors and cell senescence markers and impaired ability to form a normal differentiated epithelium. There have been no published data and no detailed assessment of lung function in HIV^−^ individuals taking PrEP. It deserves further investigations to examine PrEP toxicity on BC and the effect of PrEP on BC and other lung cell functions.

Together, our pilot study with limited number of PLWH suggest that HAART contributes to BC senescence in HIV individuals. There is a need for a large-scale study to confirm our observations. Other possible mechanisms of HIV-associated lung aging include direct effect of HIV or viral proteins and opportunistic infections^2,23,47^. Our prior studies demonstrated that HIV can induce BC to express inflammatory mediators and MMP-9^27,28^. We demonstrate that disordered epithelium observed in PLWH is closely associated with BC senescence due to the adverse effects of HAART on BC. These findings provide an explanation of higher incidence of age-related COPD among HIV population in the era of HAART.

## Supplementary Information

Supplementary Information.
