# Supplementary material for: Impaired differentiation of small airway basal stem/progenitor cells in people living with HIV
Source: Sci Rep. 2022 Feb 22;12:2966. doi: 10.1038/s41598-022-06373-7 (PMC8864005; doi:10.1038/s41598-022-06373-7)

Supplemental Data Cover Page

Impaired Differentiation of Small Airway Basal Stem/Progenitor Cells in People Living with HIV

Nancy P.Y. Chung[1], K.M. Faisal Khan[1], Mirko Andreoli[1], Robert J. Kaner[1,2], Sarah L. O'Beirne[1,2] and Ronald G. Crystal*[1,2]

[1]Department of Genetic Medicine

and

[2]Department of Medicine
Weill Cornell Medical College
New York, New York

Supplemental Figure Legends

Supplemental Figure 1. HAART induces BC senescence phenotypes in a dose dependent manner. BC from nonsmokers were treated with increasing doses of FTC, TDF (either alone or in combination) from 0.5 to 10 μM. BC were plated in T25 flask at density of 3000 cells/cm^2 and cultured in complete PneumaCult Ex-Plus medium containing HAART for 2 passages (passage 1: day 0 to 4; passage 2: day 5 to 14). Cells and total RNA were collected from passage 2 for assessment of (A) β-galactosidase expression (B) mitochondrial membrane potential and (C) p16 mRNA expression. β-galactosidase expression, mitochondrial membrane potential and mRNA expression of p16 of BC treated with HAART were measured as previously described. Results shown are the data from three independent experiments in panel A and panel B and four independent experiments for panel C.

Supplemental Figure 2: Removal of HAART could not reverse senescent phenotype in pre-HAART exposed BC. BC from nonsmokers were treated with FTC, TDF (either alone or in combination) at 1 and 5 μM. DMSO (0.05%) was used as control. BC were plated in T25 flask at density of 3000 cells/cm^2 and cultured in complete PneumaCult Ex-Plus medium containing HAART for 2 passages (passage 1: day 0 to 4; passage 2: day 5 to 14). On day 14, BC were plated in complete PneumaCult Ex-Plus medium without HAART at density of 3000 cells/cm^2 for additional 14 days. Cells were harvested and assessed for (A) β-galactosidase expression (B) mitochondrial membrane potential and (C) p16 mRNA expression.

Supplemental Figure 3. Immunofluorescence of mouse IgG_1 isotype for tubulin IV (ciliated cells) and MUC5AC (secretory cells) in HIV^- (upper panel) and PLWH SAE BC-derived airway epithelium (lower panel). ALI sections were co-stained with DAPI to reveal the whole airway epithelium sections. Bar = 20 μm.

Supplemental Figure 4. Immunofluorescence of rabbit IgG for MUC5B (secretory cells) in HIV$^-$ (upper panel) and PLWH SAE BC-derived airway epithelium (lower panel). ALI sections were co-stained with DAPI to reveal the whole airway epithelium sections. Bar = 20 µm.

Supplemental Figure 1

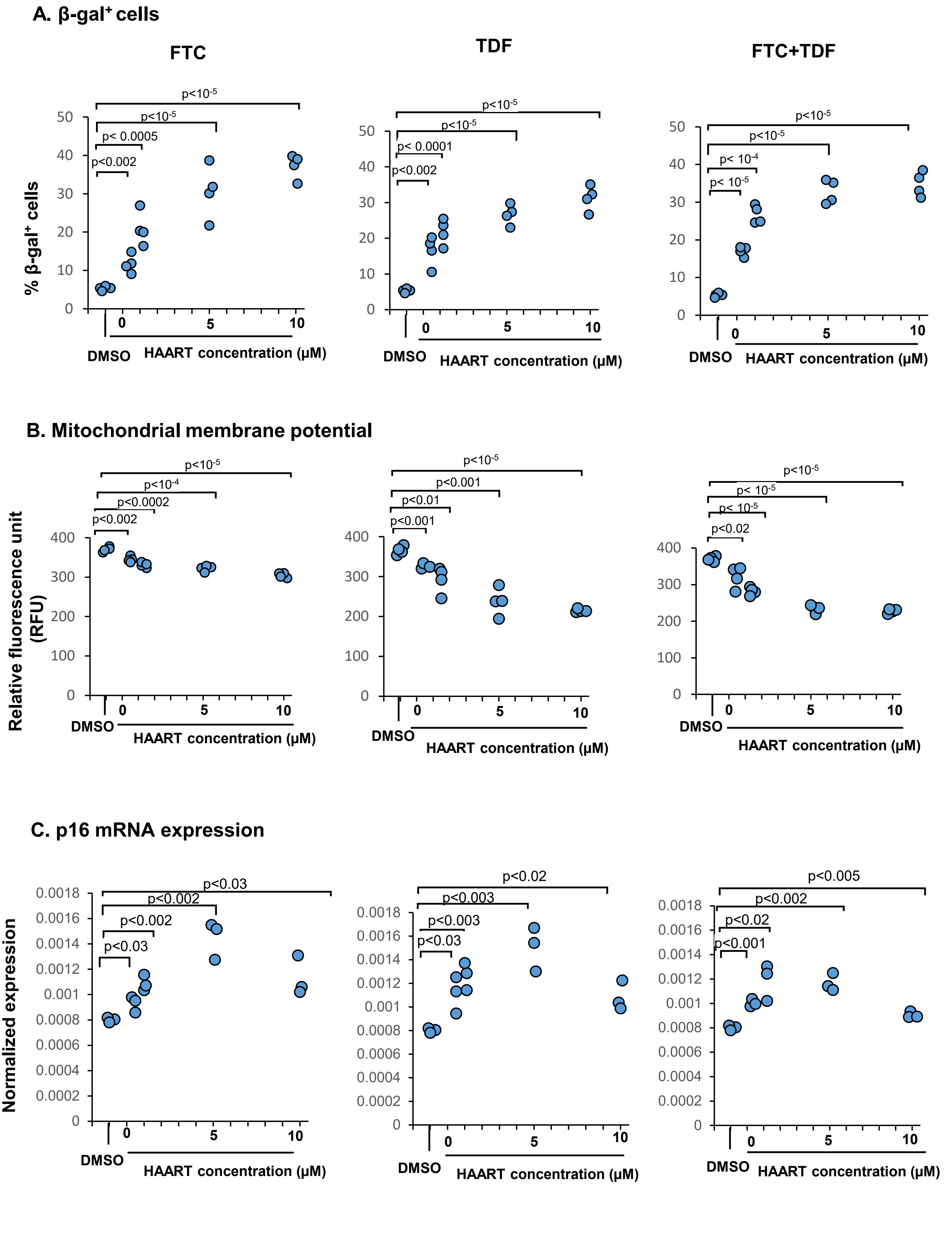

Supplemental Figure 2

A. β-gal⁺ cells

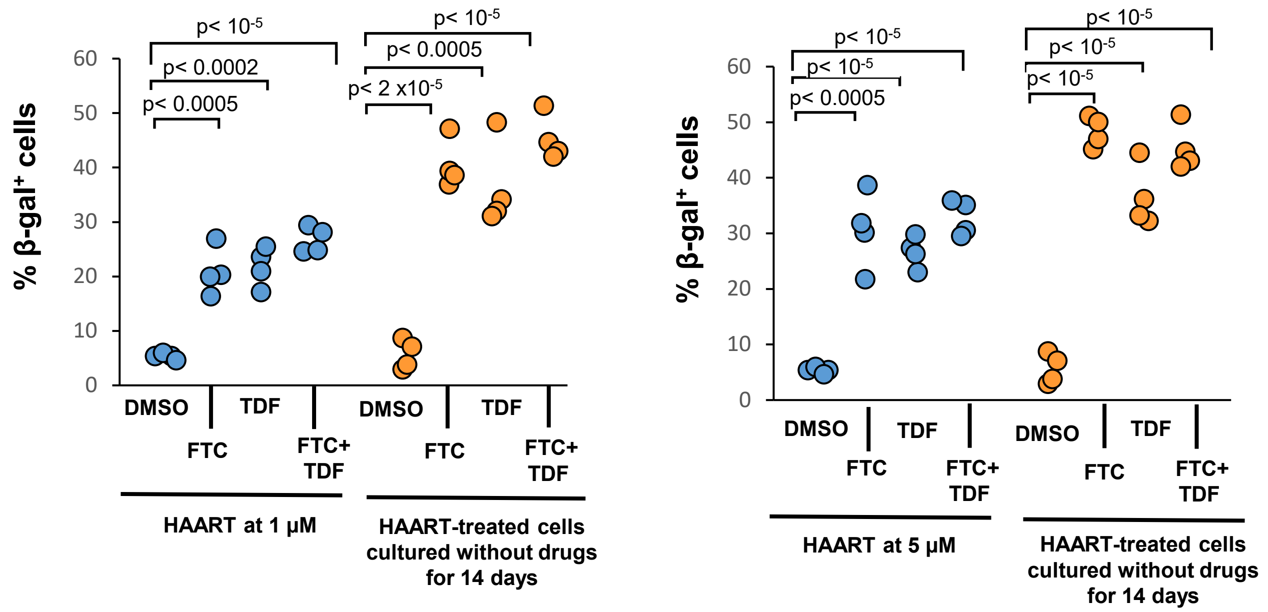

B. p16 mRNA expression

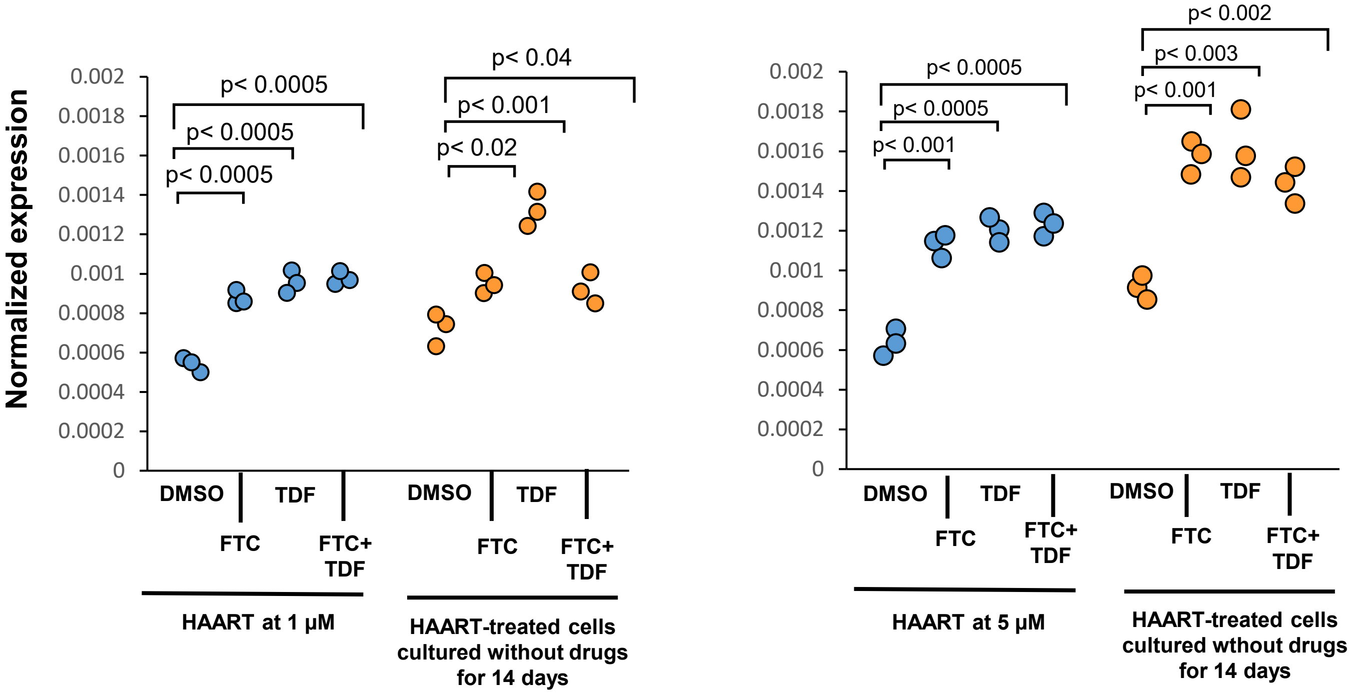

Supplemental Figure 3

A. HIV^{-} SAE BC

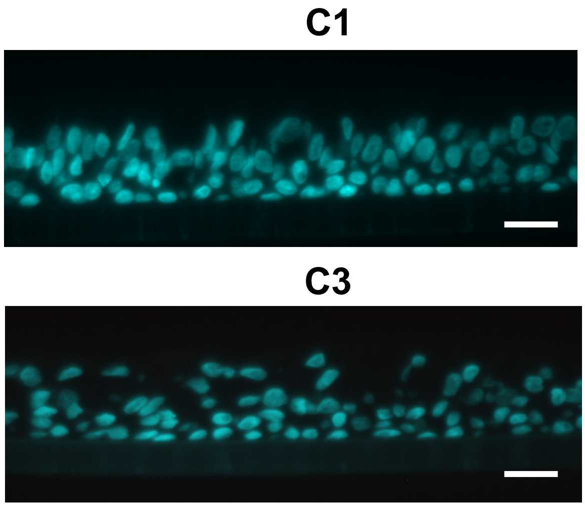

B. PLWH SAE BC

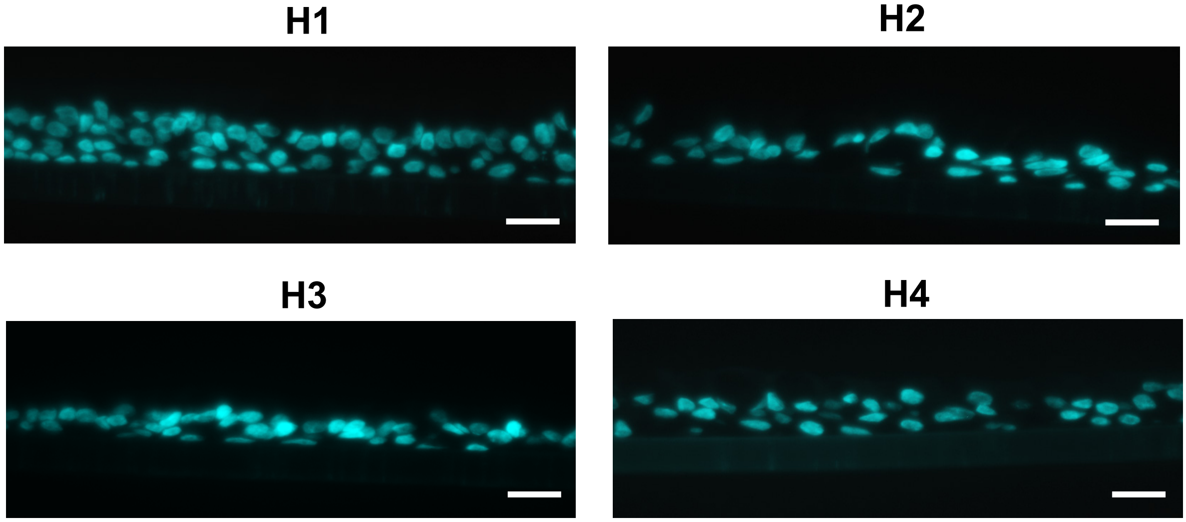

Supplemental Figure 4

A. HIV^{-} SAE BC

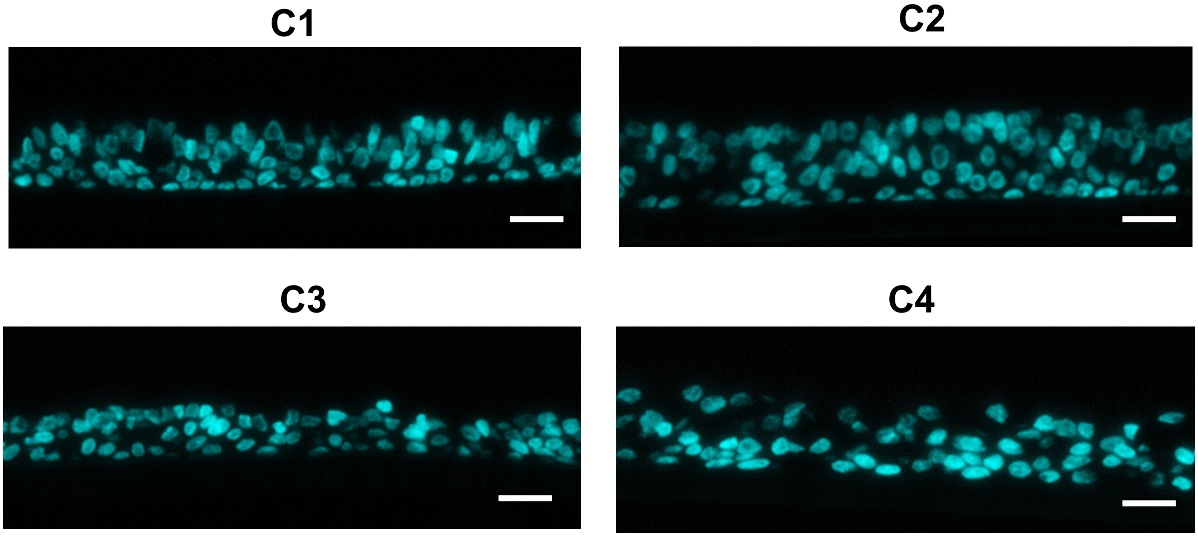

B. PLWH SAE BC

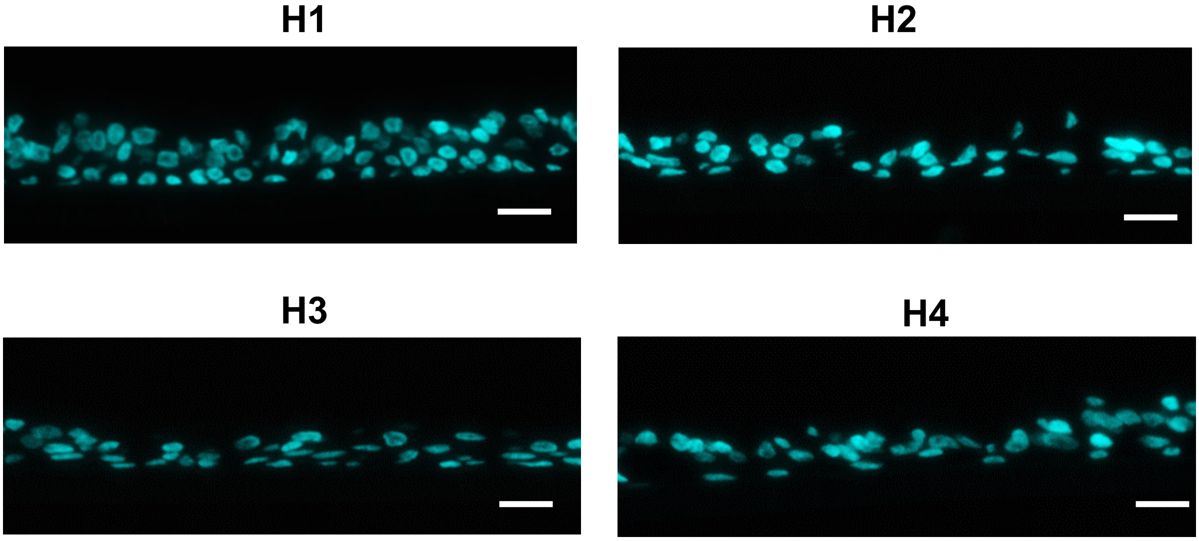

Supplement: Supplementary file 1 — Supplementary Information. [file 41598_2022_6373_MOESM1_ESM.pdf]
